# The maintenance and monitoring of perioperative blood volume

**DOI:** 10.1186/2047-0525-2-9

**Published:** 2013-05-07

**Authors:** Takehiko Iijima, Birgitte Brandstrup, Peter Rodhe, Audrius Andrijauskas, Christer H Svensen

**Affiliations:** 1 Department of Perioperative Medicine, Division of Anesthesiology, School of Dentistry, Showa University; 2 Department of Surgical Gastroenterology, Hvidovre University Hospital, Copenhagen, Denmark; 3 Karolinska Institutet, Department of Clinical Science and Education, Section of Anesthesiology and Intensive Care, Södersjukhuset, Stockholm, Sweden; 4 Clinic of Anesthesiology and Intensive Care, Vilnius University, Vilnius, Lithuania

**Keywords:** Fluid therapy, Blood volume, Blood volume assessment, Monitoring, Fluid responsiveness

## Abstract

The assessment and maintenance of perioperative blood volume is important because fluid therapy is a routine part of intraoperative care. In the past, patients undergoing major surgery were given large amounts of fluids because health-care providers were concerned about preoperative dehydration and intraoperative losses to a third space. In the last decade it has become clear that fluid therapy has to be more individualized. Because the exact determination of blood volume is not clinically possible at every timepoint, there have been different approaches to assess fluid requirements, such as goal-directed protocols guided by invasive and less invasive devices.

This article focuses on laboratory volume determination, capillary dynamics, aspects of different fluids and how to clinically assess and monitor perioperative blood volume.

## Review

Fluid therapy is a routine part of intraoperative anesthetic and surgical practice, and there is increasing evidence that fluid therapy can affect perioperative outcomes [1-5]. The goals of perioperative fluid management are to restore and maintain blood volume, secure adequate perfusion to pertinent tissues, and avoid excessive administration of fluids. Traditionally, fluid therapy consisted of infusing large volumes of crystalloids, which often resulted in postoperative body weight gain [6]. This treatment was based on the premise that patients were hypovolemic due to fasting and bowel preparation. Furthermore, it was believed, based on questionable tracer methods, that surgery caused extracellular contraction that needed replacement [7-9]. In recent years, fasting rules and patient preparation have changed and the idea that one must replace fluid allocated to the third space has mostly been abandoned [10]. Care providers are also now more prone to administer vasopressors instead of fluids to offset the hypotension caused by general or neuraxial anesthesia [11-13]. Several randomized clinical trials have tested the efficacy of different fluid protocols [14-16], and many protocols aim to achieve either zero fluid balance or a near maximum stroke volume using both invasive and minimally invasive devices [17].

This article focuses on laboratory volume determination, capillary dynamics, different fluids and how to clinically assess and monitor perioperative blood volume.

### Body fluid compartments

**Figure 1:**
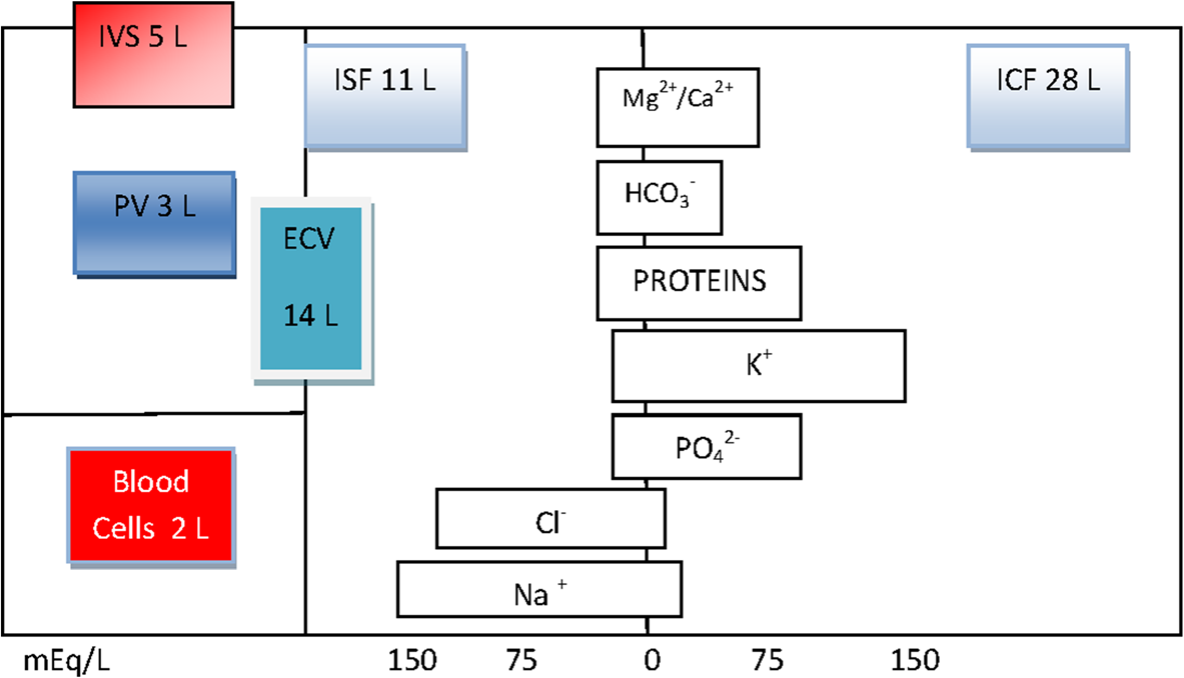
**Body fluid compartments. **Amounts in liters. mEq/L is milliequivalents per liter. Reproduced with permission [19]. ECV: extracellular volume (PV + ISF); ICF: intracellular fluid space; ISF: interstitial fluid space; IVS: intravascular space; PV: plasma volume.

The amount of water found in the body, total body water (TBW), can be assessed indirectly by any method capable of measuring body fat or fat-free mass such as hydrodensitometry, dual-energy x-ray absorptiometry or total body potassium levels. The amount of body fat thus influences the TBW: the more fat gives relatively less water [18]. The anatomical view of fluid distribution is usually represented as distinct compartments with fixed volumes. TBW comprises approximately 60% of the lean body mass, or the equivalent of 42 L in a normal-sized adult man. This volume can be subdivided into an intracellular fluid space (ICF), which consists of approximately 28 L, and an extracellular fluid volume (ECV), which occupies approximately 14 L. The ECV consists of the plasma volume (PV, 3 L) and the interstitial fluid space (ISF, 11 L). The blood volume (BV, 5 L) consists of the PV and the red cell mass or blood cells (2 L). The red blood cell mass is usually expressed as a percentage of the total blood volume and is referred to as the hematocrit (Figure 1). The osmolality in the ECV is 290 mosmol/kg. The ECV and ICF differ primarily in their concentrations of sodium (Na^+^) and potassium (K^+^). The high Na^+^ (142 mmol/L) and low K^+^ (4.3 mmol/L) concentrations in the ECV are responsible for maintaining the ECV, while the high K^+^ (139 mmol/L) and low Na^+^ (12 mmol/L) concentrations in the ICF are required for maintaining an electric potential across cell membranes. The capillary endothelial wall, however, is readily permeable to electrolytes. Oncotically active molecules together with an intact wall are necessary to create and maintain a colloid osmotic pressure (COP) of 25 mm Hg inside the vascular lumen. Because the interstitial COP is approximately 5 mm Hg, the intravascular volume remains intact. This will be discussed further in the section on the capillary wall and the glycocalyx.

### Estimation of body compartment volumes

TBW can be estimated with isotopically labeled water using deuterium or tritium. The ECV can be estimated using isotopes of bromide (^82^Br^-^) or sulfate (^35^SO_4_^2-^) and the ICF is obtained as the difference between TBW and the ECV. The PV can be estimated using either isotope-labeled albumin or different dyes. The red cell mass, or the hematocrit, can be estimated using ^51^Cr-labeled red cells. Together the PV and the hematocrit make up the total BV. The ISF is the result of subtracting the BV from the ECV. These are static measurements, with isotopes taking different times to equilibrate and can hardly be used for clinical purposes.

#### Plasma volume

The PV can be estimated using radio-iodinated serum albumin (RISA) with the ^125^I isotope [19,20]. The drawback of this method, besides its high level of radioactivity, is that albumin leaks through the capillaries into the interstitial fluid and this tends to give an overestimation of the distribution volume. Variation coefficients are between 3% and 4% [21]. Other commonly used agents for measuring the PV are Evans Blue, a dye that binds to albumin, and indocyanine green (ICG), a dye that binds to globulin [22]. The main advantage of these dyes is that they are non-radioactive and can achieve the same level of precision as that obtained with ^125^I, although they are not without their own drawbacks.

#### Blood volume

BV is defined as the total volume of blood in the body, and includes blood stored in the spleen, liver and bone marrow. Of the total circulating BV, 70% to 80% is contained in the venous circulation and in the visceral organs in particular. In 1854, Welcker bled animals to death, washed out the blood vessels and determined the hemoglobin content of the blood that had been obtained. He concluded that the blood constituted one-thirteenth of the total body weight in mammals. Many textbooks state that BV is approximately 75 mL/kg for adult men and 65 mL/kg for adult women, or 7% to 8% of body weight [23], and that the BV is slightly higher per kilogram in younger subjects [24]. Formulas based solely on body weight, however, can be misleading. Nadler included height in the estimate defining formulas that have been used frequently [23]. Prediction of BV based on body surface area may be more accurate in that this method compensates for differences in adiposity, and the International Council for Standardization in Haematology recommends formulas based on this value [25].

Blood volumes vary considerably, both between individuals and within the same individual [26,27]. Guyton and Hall showed that BV varies between individuals depending on gender and weight [28]. Furthermore, BV can vary within an individual according to oxygen consumption, as has been shown in sports medicine [29] and Iijima and co-workers used ICG to show that BV varies when patients are subjected to anesthesia [30].

Hemorrhage consists of both red cells and plasma water. It is compensated for by an initial resetting of the precapillary and postcapillary sphincters leading to the transcapillary refill state that transports fluid and protein from the interstitial space to the intravascular space [31]. Furthermore, cells become resistant to insulin resulting in hyperglycemia [32]. This causes an osmotic gradient and water flows through the cell membranes to maintain the BV while albumin is transported from the interstitium to the intravascular space through lymph channels [32-35]. Jacob *et al*. found that BV did not change significantly after fasting [13]. Previous work that has examined changes in fluid spaces following thoracic surgery has shown that there is a postoperative decrease in BV of 6% to 19% compared to preoperative values [36-39]. Results from abdominal surgery, in contrast, have been inconsistent. Shires *et al*. found that postoperative BV decreased compared to the preoperative volume, but in this study the lost blood was not replaced [9]. Ariel found that the preoperative BV was higher than the BV immediately following surgery, but had returned to preoperative levels the following morning [40]. In abdominal and orthopedic surgery, BV does not change significantly from the preoperative value [41-48]. All the above-mentioned trials of patients undergoing thoracic, abdominal or orthopedic surgery used ^51^Cr to measure red cell volume and albumin labeled with ^131^I or ^125^I to measure plasma volume. Rehm and co-workers, using a dye method for measurement, found that the PV was unchanged despite excessive infusions of crystalloid, and they confirmed that, for patients undergoing surgery for ovarian cancer, albumin leaves the circulation in ways other than direct blood loss [48].

#### Hormones in control of regulation of volume

BV and the volumes of various other compartments are also controlled by several hormones such as antidiuretic hormone (ADH), catecholamines, the renin-angiotensin-aldosterone system (RAAS), natriuretic peptides (ANP), erythropoietin and locally active neurohormonal factors. These hormones are secreted in response to the stimulation of sensors located in the kidneys and other sites such as the atria and ventricles of the heart. The distribution of water between the intravascular and extravascular spaces is regulated by modified Starling forces in combination with alteration of the vascular endothelial glycocalyx lining [15]. The sympatho-adreno-medullary axis and the hypothalamic periventricular nucleus are activated by increases in stressful stimuli [49]. The former facilitates the release of catecholamines and activates the sympathetic nervous system and the latter increases the secretion of cortisol and adrenocorticotropic hormone. These signals trigger the anterior pituitary gland to stimulate ADH secretion, which is the most potent hormone for maintaining fluid homeostasis. Catecholamines have an impact on the heart and on the regulation of peripheral vessels. Iijima *et al*. described a pediatric patient with pheochromocytoma in whom the BV increased by more than 1.2 L, from a baseline value of 2.83 L, after resection of the tumor [50]. Such catecholamine-dependent changes in BV have been confirmed in patients with pheochromocytoma after alpha-blocker therapy. Norepinephrine is a potent α-adrenergic agonist and less potent β-vasoconstrictor, and it is used as a first-line treatment for the maintenance of blood pressure in low pressure states and septic shock [51]. The impact of norepinephrine on BV, however, is not conclusive. Shibuya *et al*. used a method with ^51^Cr-labeled erythrocytes to investigate the effect of norepinephrine infusions during minor surgical procedures. In that study, blood pressure increased by 30% and PV decreased by 12% [52]. Hifumi *et al*. examined the correlation between norepinephrine and PV in hypertensive patients with high catecholamine levels using ^131^I-labeled albumin and found that norepinephrine was correlated with a low PV [53]. However, Rector *et al*. showed that high norepinephrine levels are correlated with high BV levels in cirrhosis patients with ascites [54]. ANP is secreted primarily by ventricular myocytes in response to wall stress that has been induced by volume load and increased pressure, and ANP secretion is stimulated by the rapid infusion of a fluid bolus. Some studies have failed to find a correlation between BV and ANP [55-57], while others have shown that the secretion of ANP decreases BV probably due to a diuretic effect [58-60]. More interestingly, however, ANP induces shedding of the glycocalyx, which facilitates translocation of fluid to the interstitial space [61]. Vane *et al*. showed that isoproterenol, a non-specific β-adrenergic agonist, and dopamine, a mixed β-adrenergic and dopaminergic agonist, increased PV while phenylephrine, a pure α-adrenergic agonist, decreased PV [62]. Kinsky *et al*. showed that esmolol, a β-adrenergic antagonist, decreased PV and increased extravascular volume [63,64]. Phenylephrine is commonly used for perioperative support of blood pressure but reduces global cardiac output, increases myocardial wall stress, and increases myocardial oxygen requirements and should be reserved for hypertrophic subaortic stenosis, tetralogy of Fallot, and pregnancy where it results in less fetal acidosis compared to ephedrine [65].

### Dynamics of capillaries

**Figure 2:**
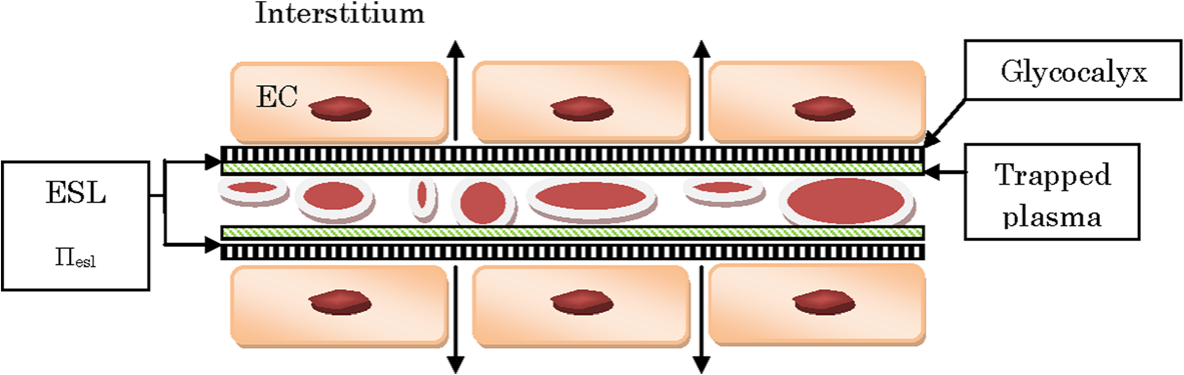
**Capillary with red blood cells and endothelial cells (EC). **The inner lining of the glycocalyx and trapped plasma together form the endothelial surface layer (ESL). A revised Starling formula would be as follows: *J*_*v*_ = *K*_*f *_([*P*_*c *_– *P*_*i*_] –Ώ[Π_esl _– Π_b_]) where *J*_*v *_is the net filtration, *K*_*f *_is the filtration coefficient, *P*_*c *_is the capillary hydrostatic pressure, *P*_*i *_is the interstitial hydrostatic pressure, Ώ is the reflection coefficient, Π_esl _is the oncotic pressure within the endothelial surface layer and Π_b _is the oncotic pressure beneath the endothelial surface layer. Within the ESL there is practically no circulation [15]. EC: endothelial cell; ESL: endothelial surface layer.

As seen in Figure 2, there are two important barriers to the distribution of fluids. First, there is the cell membrane, which keeps the interior of the cell intact. Second, there is the vascular endothelial barrier between the intravascular and the extracellular spaces. Fluid movement across the cell membrane is controlled mainly by osmotic forces and the forces governing fluid movement across the endothelial (capillary) wall were first described by Starling in 1896 [66]. These forces describe an outward movement of fluid due to the hydrostatic pressure on the arterial side of the capillary while there is a slight inward movement on the venous side. This is due to the colloid osmotic pressure within the vessels produced by the protein content of the plasma. Plasma hydrostatic and colloid osmotic pressures are counteracted by weak interstitial hydrostatic and colloid osmotic pressures. The vascular wall is permeable to water and small molecules but not to proteins or other large molecules. If isotonic solutions are given, there are no movements of fluid across the cell membrane and fluids remain in the extracellular space. Under normal conditions with an intact vascular barrier, there is a flow of fluid and albumin across the barrier with fluid and albumin returning via the lymph vessels.

#### Glycocalyx

The approach taken by Starling to describe the movement of fluids across the endothelial wall is now known to be too simplistic. The endothelium consists of a thin layer of cells with an inner fragile layer, the endothelial glycocalyx (EG), containing glycosaminoglycans (Figure 2). The glycocalyx binds plasma proteins to form the endothelial surface layer (ESL), which has a high internal oncotic pressure. The low net flux passing through the EG has low concentrations of proteins so the oncotic pressure beneath this layer is low. The EG creates a barrier that binds proteins and this prevents some erythrocytes from moving close to it. Thus, there is a zone where almost no circulation occurs and non-circulating protein-rich plasma predominates. The intravascular volume can, therefore, be considered to consist of circulating red cells and plasma volume as well as a non-circulating plasma volume. The function of the glycocalyx appears to be the maintenance of an effective colloid osmotic pressure close to 70% of the luminal osmotic pressure while keeping the colloid concentration outside the endothelium equal to that inside the lumen of the microvessel [67]. Thus, transcapillary fluid exchange seems to depend not on the difference between the hydrostatic and oncotic pressures between blood and tissue but, rather, between the blood and the small space underneath the endothelial glycocalyx but still within the lumen of the capillary. Destruction of the glycocalyx, which can occur during trauma and septicemia, will eventually cause a change in the global difference between hydrostatic and oncotic pressures and this will lead to a situation that is best described by the concepts developed by Starling. If the interstitial colloid osmotic pressure then equals that of the plasma, interstitial edema will occur. Reduction or destruction of the EG leads to platelet aggregation, leukocyte adhesion and increased permeability, all of which are hallmarks of interstitial edema. Factors that can cause destruction of the EG are proteases, ischemia/reperfusion, tumor necrosis factors, oxidized low density lipoproteins and atrial natriuretic peptide triggered by hypervolemia [15,67]. A goal for the clinician should be to maintain normovolemia during surgery to protect the EG and minimize shifts to the interstitium. Factors that have been shown experimentally to protect the endothelium are hydrocortisone, antithrombin and sevoflurane [68-70].

To determine fluid responsiveness during different conditions, it is important to understand the impact of different fluids. Over the past several decades, there has been a debate as to whether clinicians should use crystalloids or colloids. This disagreement is based mostly on tradition and local agreement on how fluid deficits should be handled and seldom on an understanding of the reason for the underlying pathophysiology. In deciding the best course of treatment, fluids should be considered as efficient drugs with their own unique impacts on patients in regard to their pharmaceutical properties, indications, contraindications and side effects.

### Different fluids

#### Crystalloids

Crystalloids have a long history of use in clinical settings. In the 1830 s saline solutions were occasionally given during the cholera outbreak in the UK. In 1881, Landerer is credited with having established the technique of giving crystalloids in clinical practice. Ringer published groundbreaking papers in 1882 verifying the importance of certain ions such as calcium and ammonium to make saline solutions more physiological. Hartmann later added buffered ions to Ringer’s solution to offset its acidity.

Crystalloids are defined as solutions containing molecules smaller than 30 kDa and thus lacking oncotic components. When glucose is administered, the glucose is metabolized in the liver leaving only water to be distributed to the ECV and ICF, and only 7% of the infused fluid will remain in the intravascular space (IVS). Isotonic crystalloids are distributed within the ECV and urine production and insensible perspiration are the primary avenues by which water is lost from the ECV. Insensible perspiration does not amount to more than 0.5 mL/kg/h in awake adults [71]. Isotonic crystalloids, preferably in a balanced form to avoid acid–base disorders [15], should theoretically be ideal for replacing losses from the ECV. Because there is a 1:5 volume ratio between the plasma space and ISF, it is estimated that 20% to 25% of an infused crystalloid will reside in the plasma space. The 1:5 distribution may only occur after a fluid bolus has been entirely equilibrated throughout the ECV during normal conditions, and provided that it still resides in the body and has not been partially eliminated. This means, however, that a crystalloid load may initially show a substantial volume effect during an ongoing infusion [72]. The hydration status of the body also influences the distribution of crystalloid solutions. This phenomenon is used in the recently introduced volume loading test, which is used to assess the hydration status of individual patients [73,74]. Tatara *et al*. showed that a 75 mL/kg crystalloid infusion after hemorrhage momentarily restored BV during the infusion. When the infusion was stopped, however, BV decreased rapidly to a volume lower than it had been before the infusion [75]. This finding supports a concept developed using homeostatic blood states theory that a physiologic target BV will change according to changes in red cell volume; for example, a decrease of red cells after hemorrhage decreases the physiologic target BV by the volume of lost red cells [73].

**Figure 3:**
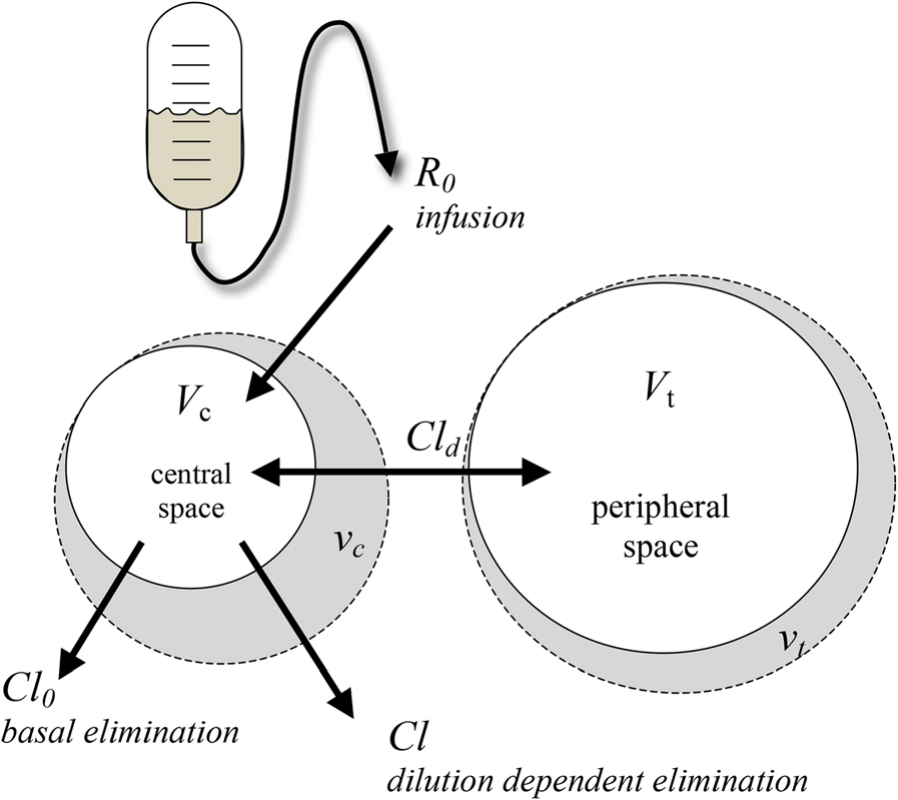
**Functional volumes in a two-fluid space model. ***R*_0 _is the infusion rate in mL/min, *V*_*c *_is the central fluid space, *v*_*c *_is the expanded central fluid space in liters, *V*_*t *_is the peripheral fluid space, *v*_*t *_is the expanded peripheral space in liters, *Cl*_0 _is the basal elimination in mL/min, *Cl*_d _is the interfluid space constant in mL/min and *Cl *is the dilution dependent elimination in mL/min.

Volume kinetics is used to study the effect of an infused bolus over time, and is similar to pharmacokinetic methods for studying drug effects. This method uses an endogenous tracer, hemoglobin, to calculate fractional changes in plasma dilution over time [76]. Repeated sampling of hemoglobin will produce a dilution curve. When such a curve has been established for an individual patient, the values can be fitted to a non-linear equation that will give estimates of the rate constants for distribution and clearance in a similar manner to pharmacokinetics. The method has been criticized, however, for being based on calculations of estimated initial values of hemoglobin and for assessing changes only in the circulating part of the BV [15]. Presumably, the method does not account for the non-circulating part of the BV. However, volume kinetics is a pharmacokinetic tool measuring functional, not anatomical, volumes and it is a useful tool to describe fluid movement between spaces. An infused bolus of a crystalloid will allocate initially in central well-perfused spaces of the body (*V*_*c*_, approximately 3 L to 4 L). This space may or may not correspond to the anatomical plasma space. Subsequently, the fluid load will be transferred to a more peripheral volume (*V*_*t*_, 6 L to 8 L), which will be smaller than the anatomical interstitial space (Figure 3). *V*_*c*_ becomes slightly larger if based on arterial rather than on venous hemoglobin values [15]. The volume of *V*_*t*_ is 6 L to 8 L in adult males weighing 70 kg to 80 kg. In contrast to earlier estimations using isotopes of sodium and bromide, volume kinetics measures changes only in spaces that are able to expand upon infusion of the fluid. There are spaces, such as bone tissue, that cannot be expanded by an infused fluid. Studying volume kinetics, it is also easy to see that under ideal conditions in awake, healthy volunteers, an infused crystalloid is readily eliminated and thus it is distributed based on a one-volume model. This is in contrast to situations during trauma and hemorrhage where the fluid has a tendency to stay in the body and a two-volume model with little or no elimination is more appropriate.

**Figure 4:**
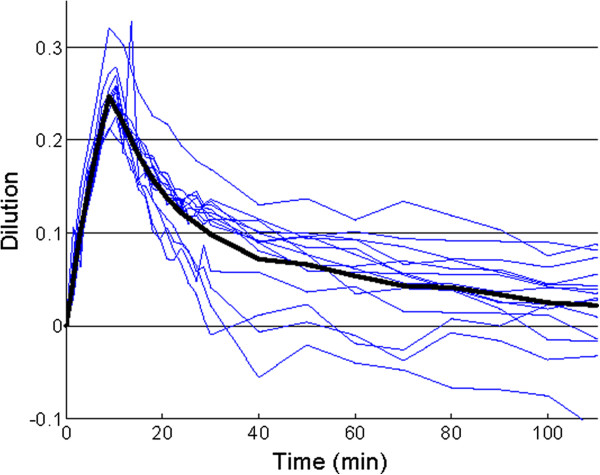
**Dilution curves (%, thin lines) for individuals. **A non-linear equation is, after iteration, fitted to the data generating a mean dilution curve (thick line) from which parameter estimates for distribution (*V*_*c*_, *V*_*t*_) and clearance (*Cl*) can be calculated [76]. The initial dilution effect of the infused bolus is approximately 25% (0.25 on the y-axis) but the effect is transient. The fluid is almost completely eliminated from the central space after 2 hours. The y-axis is the percentage dilution and the x-axis is time in minutes. In these individuals, infusion times were 10 minutes and the experiment lasted for 120 minutes.

The volume kinetics models, however, are hampered by two limitations. First, they require frequent and precise hemoglobin sampling, which is not feasible clinically. Second, they require a rather large and rapid infusion to generate substantial dilution curves. For the moment, volume kinetics is a research tool for bettering our understanding of fluid movement. In the future, accurate non-invasive measurement of hemoglobin levels may permit the clinical use of volume kinetics methods. An example of the kinetic profile of an infused crystalloid is given in Figure 4.

#### Colloids

There are a wide variety of colloids including starches, dextrans, gelatines and albumin. Artificial colloids are large macromolecules that consist of a variety of polysaccharides or polypeptides and are derived from either plant or animal sources. The artificial colloids are mainly diluted in either 0.9% saline or a balanced solution such as acetated Ringer’s solution. These are in contrast to albumin, which is a more heterogeneous colloid derived from plasma. The main reason for using colloids is to replace intravascular deficits due to events such as hemorrhage or fluid shifting [15]. Colloid infusions persist in the IVS for longer periods than crystalloid infusions because it is more difficult for large molecules to transverse an intact vascular barrier. Supposedly, the colloids can contribute to hemodynamic stability with less fluid in a shorter time. This is probably true in healthy people. In sick patients, however, the endothelial glycocalyx is often damaged leading to a leaking capillary wall [15,77]. In studies of normovolemic hemodilution, Rehm and co-workers showed that volume loading with colloids in normovolemia resulted in reduced volume persistence (68%) than if blood had already been removed (90%) [78]. Furthermore, it has been shown in clinical studies that during leakiness the volume effects of colloids are similar to that of crystalloids [79]. The ratio could actually vary from 1: 1,2-1,4 for crystalloids to colloids during these conditions [80]. The earliest evidence of renal toxicity from hydroxyethylstarch (HES) solutions came from case reports of osmotic nephrosis [81]. Several clinical studies addressing this issue have been inconclusive in critically ill patients [82-84]. Recently, two large randomized clinical studies addressed this issue in patients with sepsis and septic shock [80,85]. The Scandinavian Starch for Severe Sepsis/Septic Shock Trial (6S) was an investigator-initiated, multicenter, blinded, stratified, parallel-group clinical study with randomization. Subjects received a study fluid in a 1:1 ratio for fluid resuscitation with either HES 130/0.42 in a balanced solution or Ringer’s acetate. The study enrolled 800 patients with sepsis or septic shock in 26 centers in Scandinavia. The primary outcome was death or a need for renal replacement therapy (RRT) at 90 days after randomization. The study showed an increased mortality and also a clear renal toxicity in the HES group. The amount of trial fluid was approximately 3,000 mL in each group. However, the intervention came after the patients had been initially resuscitated, and you could argue that the patients received an unnecessary amount of HES [80]. The Crystalloid versus Hydroxyethyl Starch Trial (CHEST) enrolled 7,000 patients (including 1,937 with sepsis) and randomly assigned them to receive either HES 130/0.4 in 0.9% saline or 0.9% saline alone for all fluid resuscitation until discharge, death or 90 days. The primary outcome was death within 90 days and secondary outcomes included RRT or acute kidney injury (AKI). The inclusion criteria were quite similar to 6S but in CHEST the HES group received approximately 30% less fluid, had a faster increase in central venous pressure (CVP) and a lower incidence of cardiac failure. There was no significant difference in mortality at 90 days; however, in CHEST, HES was linked with a greater use of RRT. Of note, however, is that CHEST found a lower incidence of AKI with HES than with saline. When it comes to AKI by RIFLE criteria, CHEST favors saline as regards to creatinine while it favors HES in connection with increased urinary output [85]. However, low molecular weight colloids are widely used in goal-directed fluid protocols to reach a predetermined response for elective patients in surgical settings. Giving small aliquots of colloids up to a predetermined target volume allows fluids to be administered in a manner that more closely mimics natural physiological processes, and takes into consideration individual differences between patients [86-88]. This has improved the outcome for patients although this comparison has mostly been made with standard-of-care protocols. Whether starch solutions cause long-term renal toxicity for these patients is a question to be answered. There is currently no clinical or scientific indication that they do but there have been no long-term follow-ups from this perspective [89,90].

### Can blood volume be measured?

Estimation of BV is difficult in the clinical setting and the clinician is often faced with unspecific clinical signs of hypovolemia and dehydration. Evaluation of skin turgor, blood pressure, heart rate and urinary output are not reliable and are unsuited for guiding fluid therapy protocols. Invasive measurements such as pulmonary artery pressure, central venous pressure and blood pressure are all unreliable for detecting a fluid deficit [91]. Standard pressure preload measurements such as central venous pressure and pulmonary capillary wedge pressure may not reflect the true preload pressure [92]. Over the past several years, thermodilution and echocardiographic preload determination parameters have been shown to be superior to measurements of the pulmonary capillary wedge pressure [93,94]. In particular, the global end diastolic volume measured by transpulmonary thermodilution, the right ventricular end diastolic volume assessed by a continuous thermodilution catheter, and the left ventricular end diastolic volume assessed by echocardiography are superior methods for determining preload [92]. Many clinicians tend to look at urinary output to guide fluid therapy but this is a highly non-specific measurement. The passive leg-raising test is an easy, and probably reliable, method to test for hypovolemia [95]. A semi-automated blood volume analyzer, the BVA-100 (Daxor Corporation, NY, USA), uses a radioisotope to measure BV at the bedside. This system is used in conjunction with a single-use diagnostic kit, which uses a flow chamber and an albumin ^131^I injectate [21]. A determination of BV, PV and red cell volume with this device takes about 30 minutes and has an accuracy of ±2.5%. Another interesting method is transcutaneous pulse dye densitometry, which employs an optical probe similar to that used in pulse oximetry to measure infused intra-arterial ICG concentrations [30].

#### Zero-balanced protocols

When it became clear that aggressive fluid loading with crystalloids was harmful for perioperative fluid management [96], a more restrictive approach became apparent. In thoracic surgery, fluid restriction became standard practice, but in the general surgical population this was more dependent on the discretion of the clinician. In 2003, Brandstrup and co-workers published a randomized multicenter study comparing liberal and restrictive fluid management strategies in colorectal surgery [1]. The number of postoperative patients with complications was significantly reduced in those receiving more restrictive fluid therapies. This study looked at anastomotic leakage, wound infection, and cardiovascular and pulmonary complications, all of which were reduced when perioperative fluid was limited. Postoperative weight gain was determined to be a negative factor from these limited treatment protocols. This study has been widely cited and counts as a major landmark study in its field, but it has been pointed out that the restrictive group received more colloids compared to the saline standard-of-care group. Several studies have since addressed this topic and it can be concluded that fluid amounts in groups considered restrictive and liberal vary considerably. What is restrictive in one study can very well be liberal in another and the types of surgery also vary considerably in these studies. Some studies have been performed on day surgical cases where the endpoints (well-being, less nausea, vomiting and dizziness) are different compared to more high-risk surgical patients where length of hospital stay and mortality are more relevant as endpoints. Healthy subjects undergoing short procedures seem to benefit from a moderate infusion of a crystalloid to prevent postoperative nausea [97] while patients undergoing colorectal surgery have benefited from zero-balanced protocols with a restrictive goal-directed protocol pattern, even though the total amount of fluid could exceed that administered during day surgery protocols due to the length of surgery.

#### Individualized goal-directed therapy

**Figure 5:**
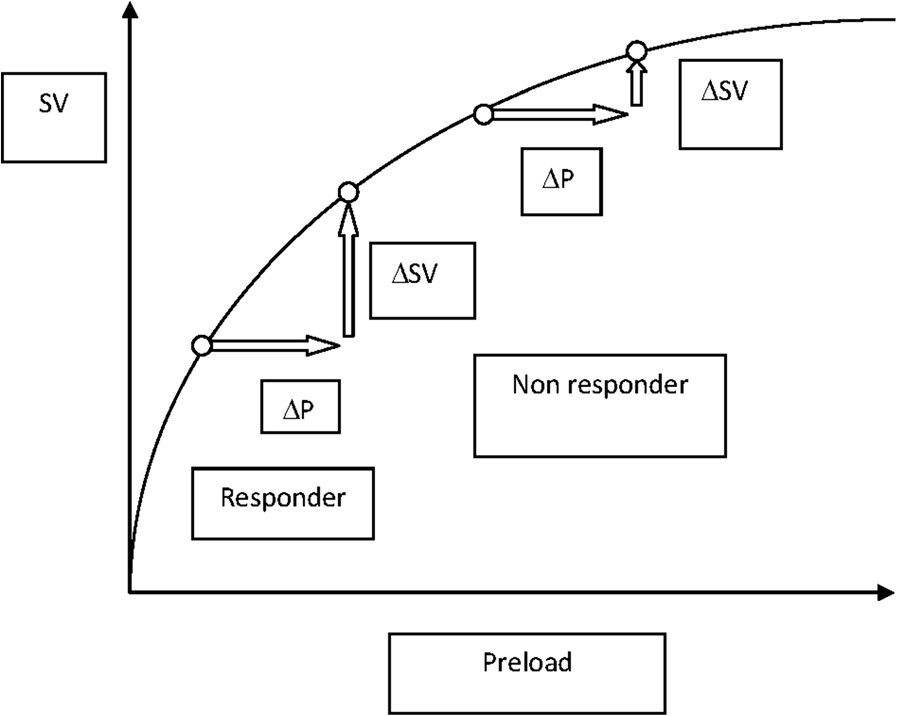
**The Frank–Starling curve showing the relationship between diastolic myocardial tension (preload) and systolic cardiac function (stroke volume). ** An increase in preload results in a corresponding increase in stroke volume. Using the curve, it is possible to analyze who is a responder and who is a non-responder to fluid therapy. P: preload; SV: stroke volume.

**Figure 6:**
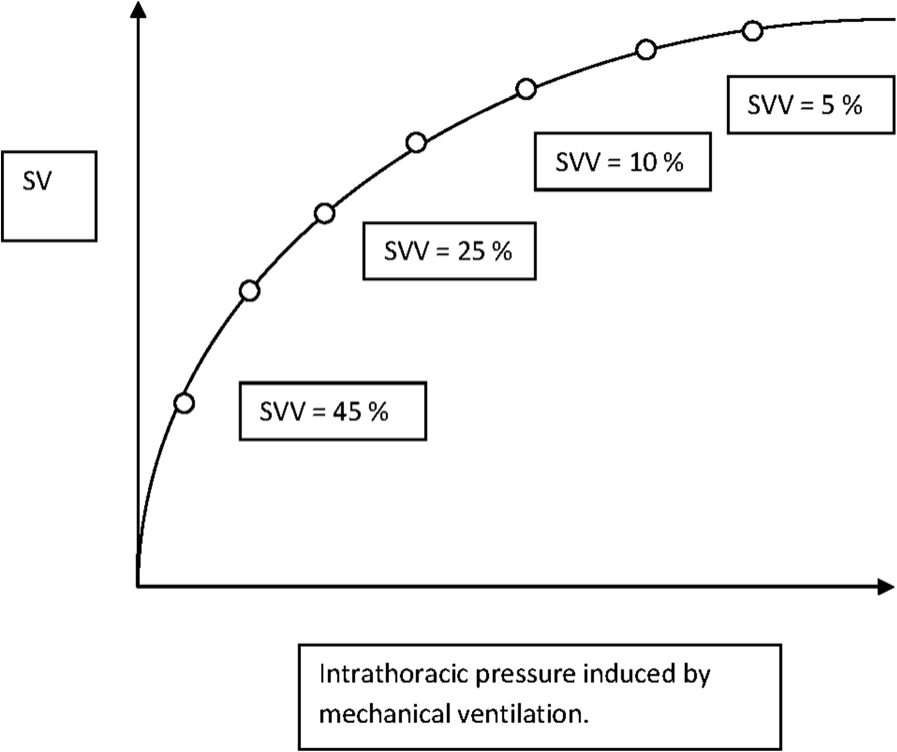
**The correlation between stroke volume variation (SVV) and the Frank–Starling curve. **Round circles indicate changes in SVV as more fluid is given. When SVV is below 10% to 12%, the patient is said to be a non-responder. SV: stroke volume; SVV: stroke volume variation.

Individualized fluid therapy is likely the best course of action because patients differ in underlying pathology, size and responsiveness to different physiological perturbations. Therefore, it would be beneficial to be able to determine the baseline hydration status for each individual. This task is, indeed, a challenge for the clinician and significant amounts of research are directed towards methods for finding the baseline hydration status of an individual on the day of surgery. Intermittent estimations of BV and the fluid status in different compartments using an experimental tracer or dilution methods are not clinically feasible. Instead, an ideal therapy would measure responsiveness, which is the deviation of target parameters induced by a fluid bolus. However, fluid responsiveness requires a dynamic assessment. The Frank–Starling curve describes the relationship between diastolic myocardial tension (preload) and systolic cardiac function (Figures 5 and 6).

An increase in preload should cause an increase in cardiac output. From the curve, it is possible to analyze which patients are responders and which are non-responders to fluid therapy in the context of cardiovascular performance [17]. Functional hemodynamic parameters such as stroke volume variation (SVV) and pulse pressure variation could possibly be used to guide fluid therapy [17,98]. Both of these parameters are dependent on the interaction between the heart and the lungs (pulse contour analysis). In a spontaneously breathing patient, inspiration will reduce the intrathoracic pressure, which will augment venous return and the preload thereby increasing stroke volume. In a mechanically ventilated patient, the opposite conditions will persist. This can be observed clinically by studying the arterial waveform during mechanical ventilation and seeing how the curve moves. The movement of the curve can actually show the degree of hypovolemia. Variations imposed on the Frank–Starling curve can result in major alterations if imposed in the lower part of the curve, but only minor alterations will occur if imposed on the flatter part.

#### Different monitoring systems

An example of an invasive device is the PiCCO (Pulsion Medical Systems, Munich, Germany), which enables continuous hemodynamic monitoring using a femoral or axillary arterial catheter and a central venous catheter. Employing patented algorithms based on the assumption that the area under the systolic part of the aortic pressure waveform corresponds to the stroke volume curve, PiCCO combines real-time continuous monitoring through pulse contour analysis with intermittent thermodilution measurement via the transpulmonary method. This device is able to give measurements of the transpulmonary cardiac output, intrathoracic blood volume, extravascular lung water and cardiac function. The latter parameters require a central venous line for injection of a cold bolus. The system, however, requires recalibrations, especially when rapid changes occur [99].

The LiDCO technique (LiDCO Ltd, London, UK) is another pulse contour method (or pulse power method), based on a purported linear relationship between net power and net flow in the vascular system provided that no major change in vascular compliance or resistance occurs. The LiDCO plus technique requires lithium dilution and gives a reliable assessment of cardiac output and stroke volume provided the restrictions mentioned above are met. The LiDCO rapid version does not require calibration and is used solely for monitoring cardiac output and stroke volume deviations from baseline [100].

The FloTrac and Vigileo (Edwards Inc., Irvine, CA) systems requires a sensor attached to an arterial line. The system continually displays cardiac output (CO) and SVV, which can be correlated to the Frank–Starling curve as seen in Figure 6. For the Vigileo system, the standard deviation of pulse pressure is correlated with a normal stroke volume based on an underlying database. Aortic impedance is also derived from historic data, but actual vascular compliance and resistance are determined using arterial waveform analysis. The device does not require calibration and is fairly easy to use but is hampered by the fact that it is less reliable when vasopressors are used [101]. Even the latest software, version 3, is not accurate when a common vasopressor such as phenylephrine is used. Furthermore, an optimal arterial signal is needed for valid cardiac output measurement. SVV measurements are also limited by the requirement of mechanical ventilation with a tidal volume of at least 8 ml/kg [91]. A new device package, the EV 1000 (Edwards Inc., Irvine, CA), combines the FloTrac system with the PreSep/PediaSet oximetry catheters to provide central venous oxygen saturation measurements (S_cv_O_2_), and gives both hemodynamic and volumetric data. The use of this device has recently been approved by the FDA in the US. All of these devices require optimal arterial signals and are unreliable when the patient has arrhythmias or is undergoing intra-aortic balloon pump treatment.

Recently, non-invasive methods have been presented such as plethysmographic systolic pressure variation and the plethysmographic variability index (Masimo Inc., Irvine, CA). These methods also require the patient to be mechanically ventilated. However, this could enhance perioperative monitoring [102].

Esophageal Doppler-guided therapy (ED) uses Doppler ultrasound technology to analyze the blood flow in the descending aorta. A single-use probe is inserted into the esophagus and positioned to measure the blood flow for each heartbeat. The waveform is then compared to biometric data and gives the stroke volume. Because the catheter is inserted in the lower esophagus, approximately 70% of the circulating blood flow is accounted for in the calculations. Various algorithms are used to measure fluid responsiveness. Most algorithms use small aliquots of colloids (2 to 3 mL/kg). The primary goal of the technique is to optimize the stroke volume according to the Frank–Starling curve. The stepwise fluid infusion is continued until the patient is considered a non-responder to fluid. Usually this happens when stroke volume or cardiac output deviation becomes <10%. This has been validated against standard-of-care protocols; reduced morbidity and reduced length of hospital stay have been shown [87,103-106]. The ED technique has been used in high-risk and colorectal surgery, but recent studies showed no differences between ED and a fixed protocol in more healthy patients or patients undergoing laparoscopic procedures [107,108].

## Conclusions and clinical guidelines

To conclude, the evidence-based recommendations are that intraoperative fluid therapy should aim to administer an adequate and timely volume-preserving flow to pertinent organs. Healthy subjects undergoing short procedures seem to benefit from a moderate infusion of a crystalloid [97]. This will offset minor postoperative complications such as nausea and vomiting.

High-risk patients, on the other hand, benefit from a carefully monitored fluid regimen based on individualized goal-directed therapy. This can be done in several diffe rent ways. The term restrictive is purposefully avoided because this is dependent on where on the Frank–Starling curve one starts the fluid regimen. It is important, therefore, to determine the patient’s responsiveness to fluid therapy. This means that fluid therapy should be moderate and individually tailored to each patient’s needs. Goal-directed therapy using the esophageal doppler technique has been widely popular, and even nationally recommended in the UK, but compared to modest regimens it has been difficult to show improvements in outcome with this technique [106]. A combination of SVV-guided fluid therapy and oximetric guidance (PreSep/S_cv_O_2_) is probably more useful for high-risk surgery provided that the arterial waveform is readable. This should be accompanied by a moderate crystalloid infusion (1 to 2 mL/kg/h).

The choice of whether crystalloids or colloids should be used is probably less important. Crystalloids should mainly be used for losses from the ECV and colloids for losses from the IVS. However, low molecular starches should be avoided when patients have damaged endothelium as in sepsis [85]. There is currently no full consensus but albumin may be a better choice for these patients [107].

## Abbreviations

6S: Scandinavian Starch for Severe Sepsis/Septic Shock Trial; ADH: Antidiuretic hormone; AKI: Acute kidney injury; ANP: Atrial natriuretic peptide; BV: Blood volume; CHEST: Crystalloid versus Hydroxyethyl Starch Trial; Cl: Dilution-dependent clearance (elimination) in mL/min; Cl0: Basal clearance (elimination) in mL/min; Cld: Interfluid space constant in mL/min; CO: Cardiac output; COP: Colloid osmotic pressure; CVP: Central venous pressure; EC: Endothelial cell; ECV: Extracellular fluid volume; ED: Esophageal Doppler-guided therapy; EG: Endothelial glycocalyx; ESL: Endothelial surface layer; HES: Hydroxyethyl starch; ICF: Intracellular fluid space; ICG: Indocyanine green; ISF: Interstitial fluid space; IVS: Intravascular space; P: Preload; PV: Plasma volume; R0: Infusion rate in mL/min; RAAS: Renin angiotensin aldosterone system; RIFLE: Risk, injury, failure, loss, end stage kidney disease; RISA: Radio-iodinated serum albumin; RRT: Renal replacement therapy; ScvO2: Central venous oxygen saturation; SV: Stroke volume; SVV: Stroke volume variation; TBW: Total body water; Vc: Central volume in L; vc: Expanded central volume in L; Vt: Peripheral volume in L; vt: Expanded peripheral volume in L.

## Competing interests

Takehiko Iijima receives lecture fees from Fresenius KABI Co. Ltd., Japan, and Otsuka Pharmaceutical Co. Ltd., Japan.

Audrius Andrijauskas received a consultant’s fee from Masimo Corp. of Irvine, CA, USA. Audrius Andrijauskas is an inventor on US Patent No. 7,788,045 B2, PCT International patent application PCT/US2011/057,362, and US provisional patent application No. 61/692,904.

Christer Svensen receives lecture fees from Fresenius KABI, Uppsala, Sweden, and has intermittently been a member of the Masimo Inc. Advisory Board.

There are no other competing interests.

## Authors’ contributions

TI came up with the original idea for this manuscript. He contributed to the design and helped to draft the manuscript. BB, PR and AA contributed to the design and helped to draft the manuscript. CS contributed to the design, helped to draft the manuscript and was also responsible for the final revision of the text. All authors read and approved the final manuscript.
